# Bacterial and fungal gut microbiota of supralittoral talitrid amphipods feeding on brown macroalgae and paper

**DOI:** 10.1371/journal.pone.0279834

**Published:** 2022-12-30

**Authors:** Seiichiro Nakamura, Junya Yumioka, Seishu Kachi, Yasunori Baba, Shigeyuki Kawai

**Affiliations:** 1 Division of Applied Life Sciences, Graduate School of Bioresources and Environment, Ishikawa Prefectural University, Nonoichi, Ishikawa, Japan; 2 Department of Environmental Science, Faculty of Bioresources and Environment, Ishikawa Prefectural University, Nonoichi, Ishikawa, Japan; 3 Laboratory for Environmental Biotechnology, Research Institute for Bioresources and Biotechnology, Ishikawa Prefectural University, Nonoichi, Ishikawa, Japan; Hokkaido University, JAPAN

## Abstract

Some macroalgae drift on the ocean and are stranded on coasts, and these stranded brown macroalgae are regarded to be degraded by organisms. Alginate is a major component of brown macroalgae. An uncovering of how carbon is cycled through brown macroalgae is needed to deeply understand coastal ecosystems. In this study, to gain insights into metabolism of brown macroalgae and alginate in the organisms, we initially confirmed that supralittoral talitrid amphipods (beach fleas or sandhoppers collected on the Shibagaki coast in Ishikawa Prefecture, Japan) fed on the brown macroalgae. We then isolated bacteria such as *Vibrio* sp. with alginate-assimilating capability from the gut of the amphipods. Metagenomic analysis of the gut of amphipods housed in several conditions (e.g. macroalgae or paper as feed, non-sterilized or sterilized environment) showed no condition-dependent compositions of bacteria and fungi, but *Vibrio* sp. were detected at high frequency, in good agreement with the isolation of *Vibrio* sp. An intervention study using antibiotics showed that amphipods fed on algae or paper at about the same rate in the presence or absence of antibiotics, and that the antibiotics had no effects on the life span. Moreover, intervention with antibiotics completely killed *Vibrio* sp. and some other bacteria, and had significant effects on the composition of the flora in the gut, with elimination of the variations observed in the guts of amphipods housed without antibiotics. These data suggest that microbes that were killed by antibiotics, including *Vibrio* sp., in the gut of talitrid amphipods are not essential for assimilation of brown macroalgae.

## Introduction

Brown macroalgae have important roles in coastal ecosystems and are also promising carbon sources for biorefinery [1–3]. It has been estimated that about 173 TgC yr^-1^ of macroalgal carbon could be sequestered [4]. Alginate is one of the major carbon sources in brown macroalgae, with levels of up to 33.5% [1]. An uncovering of how carbon is cycled through brown macroalgae is needed to deeply understand coastal ecosystems, and this requires knowledge of how brown macroalgae, and especially alginate, are degraded, metabolized, and recycled by organisms.

Some macroalgae drift on the ocean and are stranded on coasts, and these stranded brown macroalgae are degraded by microorganisms [5] and by talitrid amphipods (beach fleas and sandhoppers) [6, 7]. Talitrid amphipods are typical organisms in the supralittorial zone and are key components of the sandy beach food web [8]. These amphipods are found on the coasts in Japan, especially under brown macroalgae [9]. With regard to bacteria, *Vibrio* sp. have been identified as decomposers of alginate and producers of alginate lyases [10, 11]. Abdelrhman *et al*. suggested involvement of gut microbiota in carbon source assimilation in talitrid amphipods [8].

To understand further how brown macroalgae, and especially alginate, are degraded and metabolized by talitrid amphipods, in the current study we first confirmed that talitrid amphipods fed on stranded brown macroalgae, which indicates that talitrid amphipods degrade and metabolize the brown macroalgae in their guts. To understand the mechanism of degradation and metabolism of the brown macroalgae, we focused on microbes in the amphipod gut, initially by isolating alginate-assimilating bacteria, and then analyzing microbiota in the gut of amphipods housed under several conditions with or without antibiotics.

## Materials and methods

### Feeding experiments

Talitrid amphipods (beach hoppers), sand and brown macroalgae (gulfweed) that had been stranded on the beach were collected at a sandy beach (a non-prohibited area) on the Shibagaki coast (36°57’ 08.9" N, 136°45’ 47.2" E) in Ishikawa Prefecture, Japan, on July 3, 2018 and Oct. 3, 2018. Beach hoppers were housed in a tank (W 27 cm × D 17 cm × H 17 cm) containing sand on which the brown macroalgae or paper (Kimwiper, Wiper S-200, Nippon Paper Crecia, Tokyo, Japan) were placed. To avoid the amphipods hopping out, the tank was covered with a net, in addition to a lid. Similar tanks without beach hoppers were used as negative controls. The tanks were kept in a room at 25°C. All macroalgae and sand used in the study were collected on the Shibagaki coast.

### Isolation of alginate-assimilating bacteria from the gut of talitrid amphipods

NTA medium consisted of 0.5% sodium alginate (1000 cps, Naclai tesque, Kyoto, Japan), 3.0% NaCl, 0.05% MgSO_4·_7H_2_O, 0.2% NaNO_3_, 0.1% K_2_HPO_4_, 0.05% KCl, 0.001% FeSO_4·_7H_2_O. NYTA was NTA containing 0.5% yeast extract (Nacalai tesque), and TA medium was NTA medium without NaCl. NT medium is NTA medium without sodium alginate. These media were solidified with 2.0% agar if needed. Cultivations of microbes were conducted at 25°C. When liquid medium was used, cultivation was conducted aerobically with PersonalLt-10F (Taitec, Saitama, Japan) at 145 strokes per minute (spm).

Guts were isolated from talitrid amphipods that had fed on brown macroalgae, using a sterilized tweezer under a microscope (Leica, EZ4D). The gut was suspended in 1.0 mL of sterilized 0.9% NaCl and the suspension (100 μL) was directly spread on solid NTA or NYTA medium. For the enrichment culture, the suspension (100 μL) was inoculated into 3.0 mL liquid NTA or NYTA medium, aerobically cultivated for 3–5 days, and diluted 100- and 10,000-fold in sterilized 0.9% NaCl. The diluted solution was spread on solid NTA or NYTA medium. Colonies were purified using the same solid medium. The alginate-degrading activity of the isolated strain was visualized with Gram’s iodine (Sigma, HT902) [12] or TLC [13]. Mixtures of DEH and oligoalginates were prepared through digestion of sodium alginate by exo-type (Atu3025) and endo-type (A1-I) alginate lyase [14]. For the Gram’s iodine method, strains were cultivated in NTA liquid medium for 2 days, washed with 3.0% NaCl, and adjusted to OD_600_ of 1.0 with 3.0% NaCl. This suspension (5.0 μL) was spotted onto NTA solid medium, further incubated for 3 days, and stained with Gram’s iodine. For TLC, strains were cultivated in NTA liquid medium containing 2.0% sodium alginate for appropriate periods. The supernatant (5.0 μL) of the culture was spotted on HPLC Silica gel 60F254 (Merck), developed with solvent (1-butanol/acetate/water = 3:2:2), and visualized by heating after spraying with 10% sulfuric acid in ethanol.

The primers used in the study are listed in Table 1. To identify the isolated bacteria, 16S rRNA was amplified with colony PCR using primers 27F and 1492R and KOD FX Neo (Toyobo, Osaka, Japan), purified, and sequenced with each of primers 27F and 1492R. Sequence data were analyzed using EzBiocloud [15].

**Table 1:** Primers used in the study.

Name	Sequence
27F	AGAGTTTGATCCTGGCTCAG
1492R	GGTTACCTTGTTACGACTT
341F ^a^	ACACTCTTTCCCTACACGACGCTCTTCCGATCTCCTACGGGNGGCWGCAG
805R ^a^	GTGACTGGAGTTCAGACGTGTGCTCTTCCGATCTGACTACHVGGGTATCTAATCC
gITS7 ^a^	ACACTCTTTCCCTACACGACGCTCTTCCGATCTGTGARTCATCGARTCTTTG
ITS4 ^a^	GTGACTGGAGTTCAGACGTGTGCTCTTCCGATCTCCTSCGCTTATTGATATGC
1389F ^a^	ACACTCTTTCCCTACACGACGCTCTTCCGATCTTTGTACACACCGCCC
1510R ^a^	GTGACTGGAGTTCAGACGTGTGCTCTTCCGATCTCCTTCYGCAGGTTCACCTAC
2nd Forward ^a^^,^ ^b^	AATGATACGGCGACCACCGAGATCTACAC[XXXXXXXX]ACACTCTTTCCCTACACGACGC
2nd Reverse ^a^^,^ ^b^	CAAGCAGAAGACGGCATACGAGAT[YYYYYYYY]GTGACTGGAGTTCAGACGTGTG

^a^ The adapter sequence is underlined.

^b^ XXXXXXXX: Illumina I5 index (8 bp). YYYYYYYY: Illumina I7 index (8 bp).

To measure growth (OD_600_) of the isolated bacteria, the bacteria were precultured in 3.0 mL of NTA or NYTA liquid medium, collected by centrifugation, washed twice with 3.0% NaCl, and suspended in 3.0% NaCl to reach OD_600_ of 0.10. The suspension (50 μL) was inoculated into 3.0 mL liquid medium and cultivated.

### First metagenomic analysis

Each stranded brown macroalgae (gulfweed) was considered to be a separate residence of the talitrid amphipods. Collection was performed on Oct. 17, 2019 on the Shibagaki coast. Talitrid amphipods under the macroalgae, the sand around the macroalgae, and the macroalgae itself were collected from two separate residence sites, which are referred to as brown macroalgae α and β (S1A Fig in S1 File). Amphipods from each residence were housed with the macroalgae or paper as above in a 1 L beaker containing 300 mL of sand. The beaker was covered with aluminum foil. For sterilized conditions, beakers containing sand and either algae or paper were autoclaved. The algae were dried at 60°C overnight before autoclaving. The experimental conditions were as shown in Table 2. The amphipods were washed twice in 5.0 mL of sterilized 3.0% (w/v) NaCl by vortex and 10 washed amphipods were released into each beaker.

**Table 2:** Talitrid amphipods and conditions of housing of the amphipods for metagenomic analysis.

No.^a^	Talitrid amphipods ^a^	Sex ^a^	Non-sterilized / sterilized ^b^	Feed^c^	Stranded macroalgae ^d^	Days	Antibiotics	Condition ^e^
**1**	-	-	non	algae	β	-	-	A
**2**	*P*. *joi*	M	non	algae	α	7	-	B
**3**	Juvenile	M	non	algae	α	7	-	B
**4**	Juvenile	M	non	algae	α	7	-	B
**5**	Not identified	F	non	algae	α	7	-	B
**6**	*P*. *joi*	M	stet	algae	α	14	-	C
**7**	Juvenile	M	ste	algae	α	14	-	C
**8**	Not identified	F	ste	algae	α	14	-	C
**9**	Not identified	F	ste	algae	α	14	-	C
**10**	Not identified	F	ste	algae	α	14	-	C
**11**	Not identified	F	ste	algae	α	14	-	C
**12**	*P*. *joi*	M	non	algae	β	7	-	D
**13**	*S*. *nipponensis*	M	non	algae	β	7	-	D
**14**	*S*. *nipponensis*	M	non	algae	β	7	-	D
**15**	Not identified	F	non	algae	β	7	-	D
**16**	Not identified	F	non	algae	β	7	-	D
**17**	Not identified	F	non	algae	β	7	-	D
**18**	*P*. *joi*	M	ste	algae	α	7	-	E
**19**	*S*. *sinensis*	M	ste	algae	α	7	-	E
**20**	Juvenile	M	ste	algae	α	7	-	E
**21**	Not identified	F	ste	algae	α	7	-	E
**22**	Not identified	F	ste	algae	α	7	-	E
**23**	Not identified	F	ste	algae	α	7	-	E
**24**	*S*. *sinensis*	M	ste	paper	α	7	-	F
**25**	Juvenile	M	ste	paper	α	7	-	F
**26**	*P*. *joi*	M	ste	paper	α	7	-	F
**27**	Not identified	F	ste	paper	α	7	-	F
**28**	Not identified	F	ste	paper	α	7	-	F
**29**	Not identified	F	ste	paper	α	7	-	F
**30**	*S*. *nipponensis*	M	ste	paper	α	14	-	G
**31**	*P*. *joi*	M	ste	paper	α	14	-	G
**32**	Not identified	F	ste	paper	α	14	-	G
**33**	Not identified	F	ste	paper	α	14	-	G
**34**	Not identified	F	ste	paper	α	14	-	G
**35**	Not identified	F	ste	paper	α	14	-	G
**36**	nd	nd	ste	algae	ns	14	-	H
**37**	nd	nd	ste	algae	ns	14	+	I
**38**	nd	nd	ste	algae	ns	14	+	I
**39**	nd	nd	ste	algae	ns	14	+	I
**40**	nd	nd	ste	algae	ns	14	+	I
**41**	nd	nd	ste	algae	ns	14	+	I
**42**	nd	nd	ste	paper	ns	14	+	J
**43**	nd	nd	ste	paper	ns	14	+	J
**44**	nd	nd	ste	paper	ns	14	+	J
**45**	nd	nd	ste	paper	ns	14	+	J

^a^ Sample No. 1: stranded brown macroalgae alone. Male and mature talitrid amphipods were identified as *Platorchestia joi* (*P*. *joi*), *Sinorchestia nipponensis* (*S*. *nipponensis*), and *Sinorchestia sinensis* (*S*. *sinensis*).

^b^ non: non-sterilized, ste: sterilized.

^c^ algae: stranded brown macroalgae.

^d^ ns: not specified. “α” and “β” indicate stranded macroalgae α and β from which amphipods were collected (S1A Fig in S1 File).

^e^ Condition A: Non-sterilized stranded macroalgae β.

Conditions B and D: Amphipods collected from stranded macroalgae α (B) and β (D) were housed in non-sterilized conditions with algae as feed for 7 days in the absence of antibiotics.

Conditions C and E: Amphipods collected from stranded macroalgae α were housed in sterilized conditions with algae as feed for 14 (C) and 7 (E) days in the absence of antibiotics.

Conditions F and G: Amphipods collected from stranded macroalgae α were housed in sterilized conditions with paper as feed for 7 (F) and 14 (G) days in the absence of antibiotics.

Condition H: An amphipod collected from non-specified stranded macroalgae was housed in sterilized conditions with algae as feed for 14 days in the absence of antibiotics.

Conditions I and J: Amphipods collected from non-specified stranded macroalgae were housed in sterilized conditions with algae (I) and paper (J) as feed for 14 days in the presence of antibiotics.

Talitrid amphipods that had fed on macroalgae or paper and had been housed for 7 or 14 days were observed by microscopy to identify their sex and species [9, 16] and were then sterilized in 70% ethanol for about 30 sec. The gut was isolated from each individual and metagenomic DNA was purified from the gut using a FastDNA Spin Kit For Soil (MP Biomedicals). Metagenomic (amplicon) analysis was conducted at Fasmac (Atsugi, Japan) as follows. The quality and quantity of the DNA was checked with Qubit (Thermo Fisher Scientific). 16S rRNA V3-V4, ITS2, and 18S rRNA V9 regions were amplified with 1st PCR using ExTaq HS (Takara, Otsu, Japan) and primers 341F/805R [17], gITS7/ITS4 [18], and 1389F/1510R [19], respectively (Table 1). 2nd PCR was conducted with ExTaq HS using the purified product of 1st PCR as a template and primers 2nd Forward and 2nd Reverse (Table 1). Cluster formation was conducted with a MiSeq System v2 using a MiSeq Reagent Kit v3 and PhiX Control Kit v3 according to the MiSeq Reagent Kit v3 Reagent Preparation Guide Rev.B. Sequence analysis was performed with MiSeq System v2 on MiSeq Control Software (MCS) ver. 2.4.1.3, Real Time Analysis (RTA) ver. 1.18.54, and bcl2fastq ver. 1.8.4 according to the bcl2fastq ver. 1.8.4 User Guide Rev.B. The sequence analysis base length was 300, and the sequence analysis technique was Paired end with PhiX addition. Raw paired-end reads (fastq) data were analyzed with the Qiime2 (ver. 2020.2) pipeline [20]. DADA2 was used to process the data [21]. Taxonomy was assigned to the 16S or 18S data using the SILVA reference database (silva_132_release) [22] from which the V3-V4 or V9 region was extracted using the 341F/805R or 1389F/1510R primer information. A Naive Bayes model was trained using the extracted regions. Taxonomy was assigned to the ITS2 data using the UNITE database [23]. A Naive Bayes model was trained using the database without extraction.

### Intervention study and second metagenomic analysis

Stock solutions of the antibiotics ampicillin, streptomycin, and kanamycin were prepared in water at 50, 50, and 25 mg/mL, respectively, and sterilized with a filter (pore size 0.2 μm). To determine the concentrations of antibiotics for the study, suspensions (OD_600_ of 1.0) of *P*. *homiensis* SK6416 and *V*. *chemaguriensis* SK6422 (Table 3) were prepared as above, diluted to OD_600_ of 0.1 and 0.01, and spotted onto solid NTA medium containing the antibiotics. The solid media were incubated for 4 days and stained with Gram’s iodine.

**Table 3:** Alginate-assimilating bacteria.

Strains	Top hit species ^a^	Top hit strain ^b^	Similarity (%) ^c^	Size of query (b)
SK6405 ^d^^,^ ^e^	*Paracoccus fistulariae*	KCTC 22803	97.67	1,289
SK6416 ^e^	*Paracoccus homiensis*	DD-R11	98.2	1,246
SK6401	*Vibrio algivorus*	SA2(T)	100	1,256
SK6402 ^d^^,^ ^e^	*Vibrio algivorus*	SA2(T)	100	1,363
SK6376	*Vibrio algivorus*	SA2(T)	99.93 ^f^	1,362
SK6421	*Vibrio algivorus*	SA2(T)	99.93 ^f^	1,377
SK6418	*Vibrio algivorus*	SA2(T)	99.92	1,277
SK6422	*Vibrio chemaguriensis*	SBOTS_Iso1	99.92	1,330
SK6371	*Vibrio neocaledonicus*	NC470	100	1,389
SK6415 ^e^	*Vibrio neocaledonicus*	NC470	99.85	1,363

^a, b, c^ Query sequence shows the highest hit for species^a^ and strain^b^ with similarity^c^.

^d^ SK6405 and SK6402 were isolated using NYTA medium. Other strains were isolated using NTA medium.

^e^ SK6405, SK6416, SK6402, and SK6415 were isolated in an enrichment culture.

^f^ 16S rRNA sequences were not identical.

Talitrid amphipods, sand and stranded brown macroalgae were collected on June 6, 2020 and Sep. 24, 2020. The amphipods were housed as described above, but with the following modifications. The sand and brown macroalgae were dried at 110°C and 60°C overnight, respectively. The 1 L beaker containing dried sand (300 mL) was sterilized by autoclaving. The dried brown macroalgae or paper were also separately autoclaved. The sterilized algae or sterilized paper was placed onto the sterilized beakers and sand, and then sterilized water without or with antibiotics (0.45 mg/mL streptomycin, 0.45 mg/mL ampicillin, and 0.225 mg/mL kanamycin) was added to a level of 1.0 mm above the sand surface. Ten amphipods washed with sterilized water without or with antibiotics were released into each beaker and housed for 14 days for the second metagenomic analysis (Table 2). This analysis was conducted as above. At 0, 3 and 7 days, photos were taken to observe the effects on feeding.

For isolation of microbes from the guts of talitrid amphipods housed with algae and antibiotics (condition I, Table 2), the guts were isolated and suspended in 3.0% NaCl, as described above. The suspension was spread on NTA, NYTA, or Marine broth 2216 (Difco) solid media in the presence or absence of antibiotics directly or after enrichment cultivation in the same liquid medium. When colonies appeared on solid media, the colony was purified using the same medium.

For life span experiments, talitrid amphipods, sand and stranded brown macroalgae were collected on April 14, 2021, Aug. 31, 2021, and Oct. 8, 2021. Nine amphipods washed with sterilized water and nine washed with sterilized water containing antibiotics were released into beakers (3 individuals per beaker: 3 beakers without antibiotics, 3 beakers with antibiotics) for the 1st experiment (conducted from May 27, 2021 to July 15, 2021) and 2nd experiment (June 24, 2021 to Aug. 12, 2021). A total of 27 individuals were washed and released (3 individuals per beaker: 9 beakers without antibiotics, 9 beakers with antibiotics) for the 3rd experiment (Oct. 7, 2021 to Nov. 11, 2021).

## Results and discussion

### Feeding experiments

The Shibagaki coast is a sandy beach located in Ishikawa Prefecture, Japan, facing the Japan Sea. Many brown macroalgae (gulfweed) were stranded on the beach (S1A Fig in S1 File), and many beach hoppers (talitrid amphipods) were observed under these macroalgae (S1B Fig in S1 File). The mature males of the talitrid amphipods were identified as *Platorchestia joi*, *Sinorchestia nipponensis*, and *Sinorchestia sinensis* (Table 2). Some immature amphipods (juveniles) were regarded as male due to immature morphological features (Table 2). All other amphipods were regarded as female, but their genera and species could not be identified due to the absence of distinguishable morphological features (Table 2).

**Fig 1:**
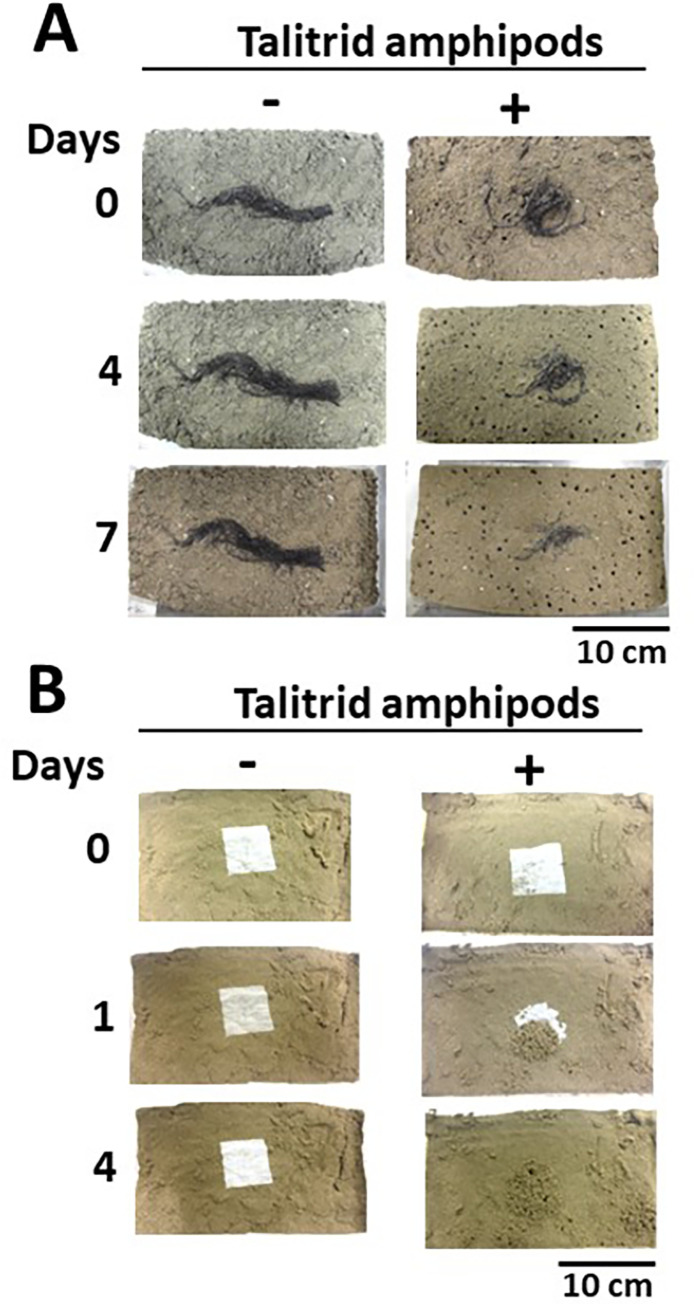
Feeding of talitrid amphipods on brown macroalgae and paper. (A) Images of stranded brown macroalgae with 30 individual amphipods (+) or without amphipods (-) after the indicated periods. (B) Images of papers with 18 individual amphipods (+) or without amphipods (-) after the indicated periods.

Nanjo previously suggested that talitridae found under stranded brown macroalgae might feed on the algae [24], but it has not been shown experimentally that amphipods in the *Talitridae* family feed on brown macroalgae. Crawley and Hyndes found that *Allorchestes compressa* (a species of amphipod in the *Dogielinotidae* family) exhibits a clear preference for brown macroalgae over red algae and seagrass as food [6]. To determine if talitrid amphipods feed on stranded brown macroalgae, the talitrid amphipods were released into tanks containing brown macroalgae or paper as feed. The talitrid amphipods fed on both brown macroalgae and paper (Fig 1). Thus, amphipods in the *Talitridae* family appear to assimilate brown macroalgae, as well as paper, through their guts.

### Isolation of alginate-assimilating bacteria from the gut of talitrid amphipods

**Fig 2:**
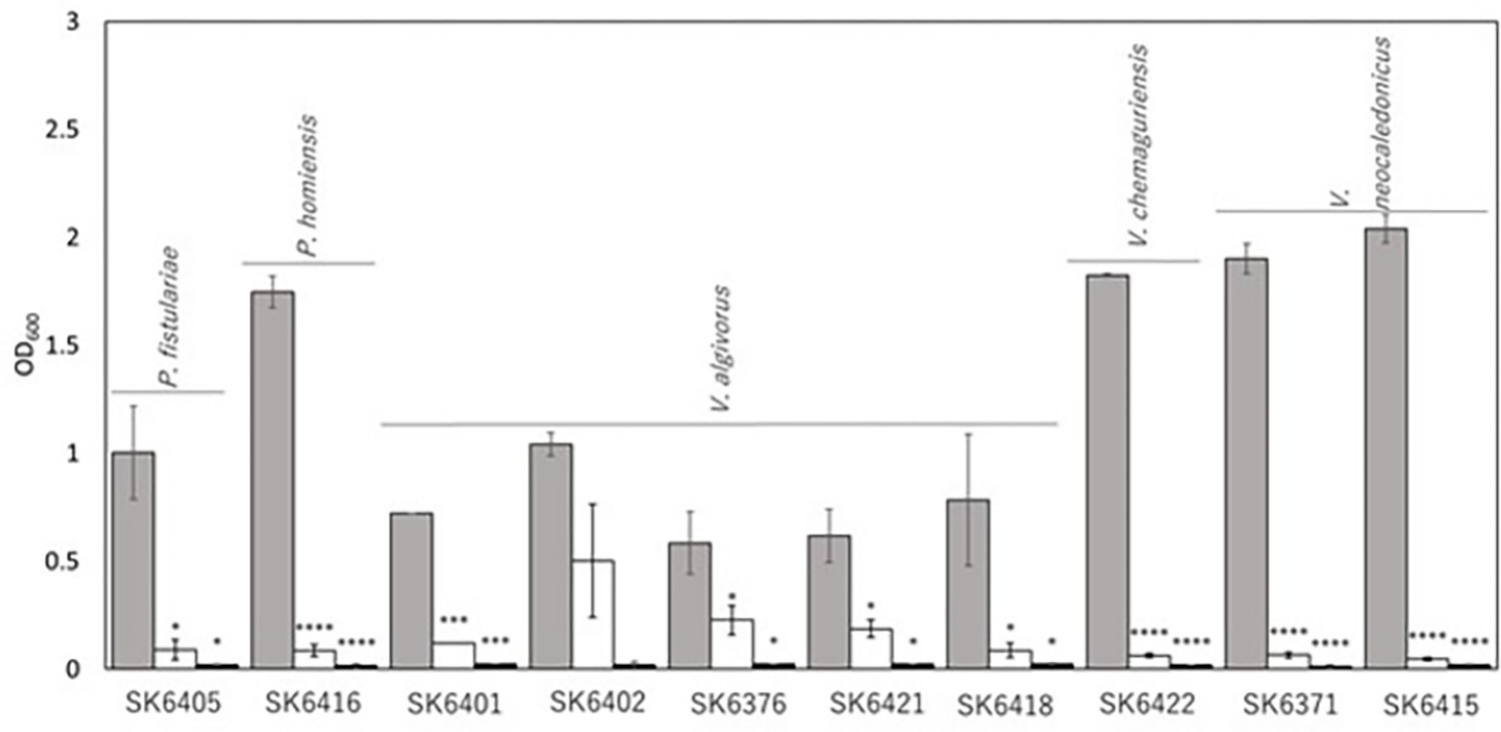
Alginate assimilation by isolated alginate-assimilating bacteria. The bacteria were cultivated in NTA (gray bar, NT plus 0.5% alginate), TA (white bar, NTA minus 3.0% NaCl), and NT (black bar, NTA minus 0.5% alginate) for 3 days. n = 3. *p < 0.05; **p < 0.01; ***p < 0.005; ****p < 0.001 vs. growth in NTA.

To address the role of the gut in assimilation of brown macroalgae, we attempted to isolate gut microbes with the capability to assimilate alginate, the main carbohydrate of brown macroalgae [1]. As shown in Table 3, 10 strains were isolated as alginate-assimilating bacteria and were identified as *Paracoccus fistulariae*, *Paracoccus homiensis*, *Vibrio algivorus*, *Vibrio chemaguriensis*, and *Vibrio neocaledonicus*, in agreement with identification of *Vibrio* sp. as decomposers of alginate and producers of alginate lyases [10, 11]. The isolated bacteria exhibited alginate-dependent growth (no growth in NT containing no organic carbon, but growth in NTA [NT plus 0.5% alginate]) and a growth defect in the absence of 3.0% NaCl (Fig 2), as well as showing alginate-degrading activity (S2 Fig in S1 File). *V*. *algivorus* SA2(T) strain has been identified as an agarose- and alginate-assimilating bacterium [25] and the genes responsible for decomposition, transport, and metabolism of alginate have been identified [26]. Doi *et al*. found that *V*. *algivorus* SA2(T) grows in the presence of 1–14% NaCl, but not in the absence of NaCl [25], while the optimal growth condition for *V*. *neocaledonicus* requires salinity of 50 g/L [27]. These findings are consistent with the properties of the bacteria isolated in this study.

### Metagenomic analysis of the gut of talitrid amphipods

Next, we examined the gut microbiota of talitrid amphipods feeding on brown macroalgae. The effects of feed (brown macroalgae or paper), sterilization (non-sterilized or sterilized environment), period of housing (7 or 14 days), residence on the sandy beach (brown macroalgae α or β), sex (male or female), and each individual on the microbiota were evaluated. Amphipods were housed under conditions B–G shown in Table 2. The gut was isolated from each individual (Nos. 2–35, Table 2), DNA was purified from each gut and metagenomic analysis was conducted. Analysis under natural conditions was performed as a control (No. 1, condition A, Table 2). A total of 2,638,615 paired-end reads with 2,318 identified features were obtained after quality filtering of 16S data. Similarly, ITS2 data gave 1,114,311 reads with 416 features and 18S data gave 89,690 reads with 74 features.

**Fig 3:**
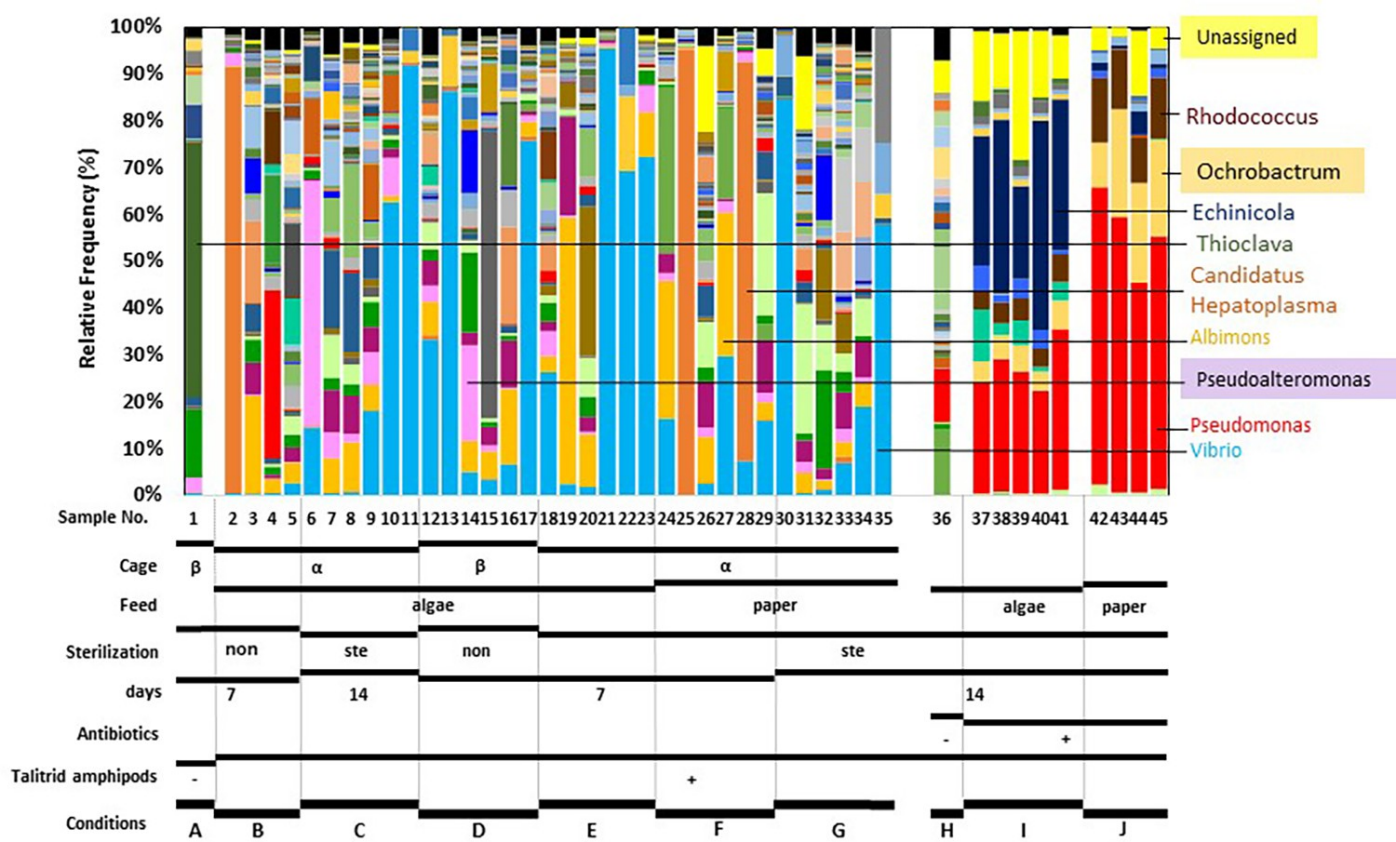
Bar plot of the relative frequency of 16S bacterial data at the genus level. The frequencies of some genera are shown in S1 Table.

**Fig 4:**
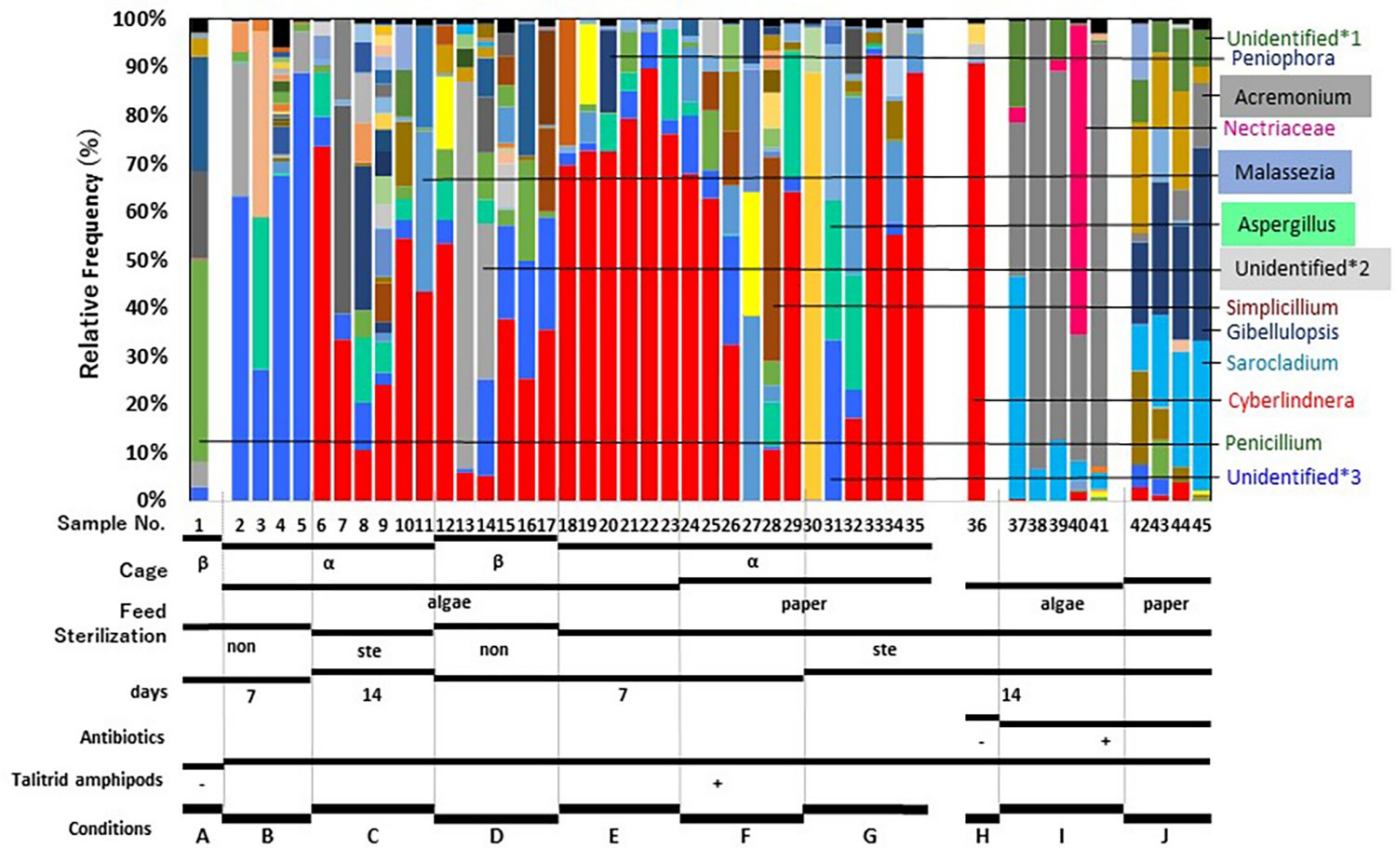
Bar plot of the relative frequency of ITS2 fungal data at the genus level. The frequencies of some genera are shown in S2 Table.

Taxonomy was assigned to the 16S (Fig 3), ITS (Fig 4), and 18S data (S3 Fig in S1 File). In the 18S data, Metazoa (Animalia) comprised the vast majority of all samples from amphipods (Nos. 2 to 35) (S3 Fig in S1 File). Accordingly, representative 18S sequences displayed similarity to each other and a Blast search showed that one of the sequences had strong similarity (99% identity, 164/165 sequences) to the partial sequence of 18S ribosomal RNA genes of *A*. *angusta* (ID: JX545346.1) and the genera *Hyalella* (e.g. MT823241.1), both of which belong to the order *Amphioda*. Thus, we concluded that almost all of the 18S data corresponded to the talitrid amphipods from which DNA was extracted, and we stopped further analysis of these data.

In contrast to the 18S data, bar plots of the relative frequency of bacterial taxa (16S data) and fungal taxa (ITS2 data) showed variations (Figs 3 and 4). In alpha- and beta-diversity analyses using the 16S data, there was no difference shown by the Shannon alpha-diversity metric (S4A Fig in S1 File) and beta diversity analysis using weighted UniFrac distances showed no clustering of samples (S5A Fig in S1 File). In bar plots of the 16S data, *Vibrio* was the major genus and was found in all 35 samples (obtained from conditions A–G) at frequencies of 0.1–95.5% (Fig 3, S1 Table). This distribution of the genus *Vibrio* was in good accord with that *Vibrio* sp. were isolated from guts of amphipods and proven to assimilate alginate (Fig 2; S2 Fig in S1 File). The genus *Vibrio* was found at low frequency (0.6%) in stranded brown macroalgae (sample no. 1, condition A), but at higher frequency in the gut of the amphipods (S1 Table). This suggests that the amphipod gut was a more suitable environment than the brown macroalgae for *Vibrio*. *Vibrio* sp. have also been detected in hadal amphipods [28].

The distribution of the genus *Vibrio* was not dependent on the feed (algae or paper) and other conditions (Fig 3, S1 Table). The genus *Paracoccus*, which was also isolated from guts of amphipods and proven to assimilate alginate (Fig 2; S2 Fig in S1 File), was also distributed, but at much lower frequencies (0.1–0.8%) in fewer samples (Nos. 6, 9, 10, 12, 18, 32, 33, and 34) (S1 Table). The genera *Pseudoalteromonas* and *Albimonas* were distributed (Fig 3, S1 Table). *Pseudoalteromonas* sp. have been reported to have alginate utilization genes [10, 29] and *Pseudoalteromonas* sp. in the gut of amphipods may assimilate alginate. *Candidatus* Hepatoplasma was also clearly distributed (Fig 3, S1 Table), but only at high frequency in 3 samples [Nos. 2 (91.2%), 25 (95.2%), and 28 (85.1%)]. This distribution of *Candidatus* Hepatoplasma is in accord with that detected in hadal amphipods [28]. The genus *Thioclava* was only found at high frequency in the stranded brown macroalgae. Collectively, condition-dependent compositions of bacteria were not observed.

Alpha- and beta-diversity analyses were conducted using the ITS2 data. Data from condition E differed from those from condition D in the Shannon alpha-diversity metric (S4B Fig in S1 File). Beta diversity analysis using weighted UniFrac distances for ITS2 data indicated that only data from condition B clustered separately from data from condition E (S5B Fig in S1 File). In bar plots, the genus *Cyberlindnera* was clearly detected in all samples except Nos 1–5, 13, 14, 27, 30, and 31 (Fig 4, S2 Table). Unidentified genera were also observed, especially in samples 2–5, 14–17, 24, and 26 (Unidentified*3) and in samples 2, 13, and 14 (Unidentified*2) (Fig 4, S2 Table).

### Effects of antibiotics

The metagenomic analysis above revealed the composition of the genera of bacteria and fungi, but no condition (i.e., feed, sterilization, or period)-dependent composition was observed. To obtain more information on the relationship between feed (algae or paper) and microbial flora, in particular for the alginate-assimilating bacteria such as *Vibrio* and *Paracoccus*, in the gut of talitrid amphipods, an intervention study using antibiotics was conducted.

Treatment with 0.45 mg/mL streptomycin, 0.45 mg/mL ampicillin, and 0.225 mg/mL kanamycin was first shown to exhibit antibiotic effects against the alginate-assimilating bacteria (*P*. *homiensis* SK6416 and *V*. *chemaguriensis* SK6422; Table 3) isolated in this study (S6 Fig in S1 File). Then, talitrid amphipods (Nos. 37–45, Table 2) were housed in the presence of the same antibiotic concentrations with algae (condition I) or paper (condition J) under sterilized conditions for 14 days (Table 2, Figs 3 and 4). As a control, the amphipods were housed with algae (No. 36, condition H, Table 2) in the absence of antibiotics under sterilized conditions for 14 days. The effects of the residence on the sandy beach, sex, and each individual on the microbiota were not included in this experiment, since these effects were unclear in the first metagenomic study described above.

**Fig 5:**
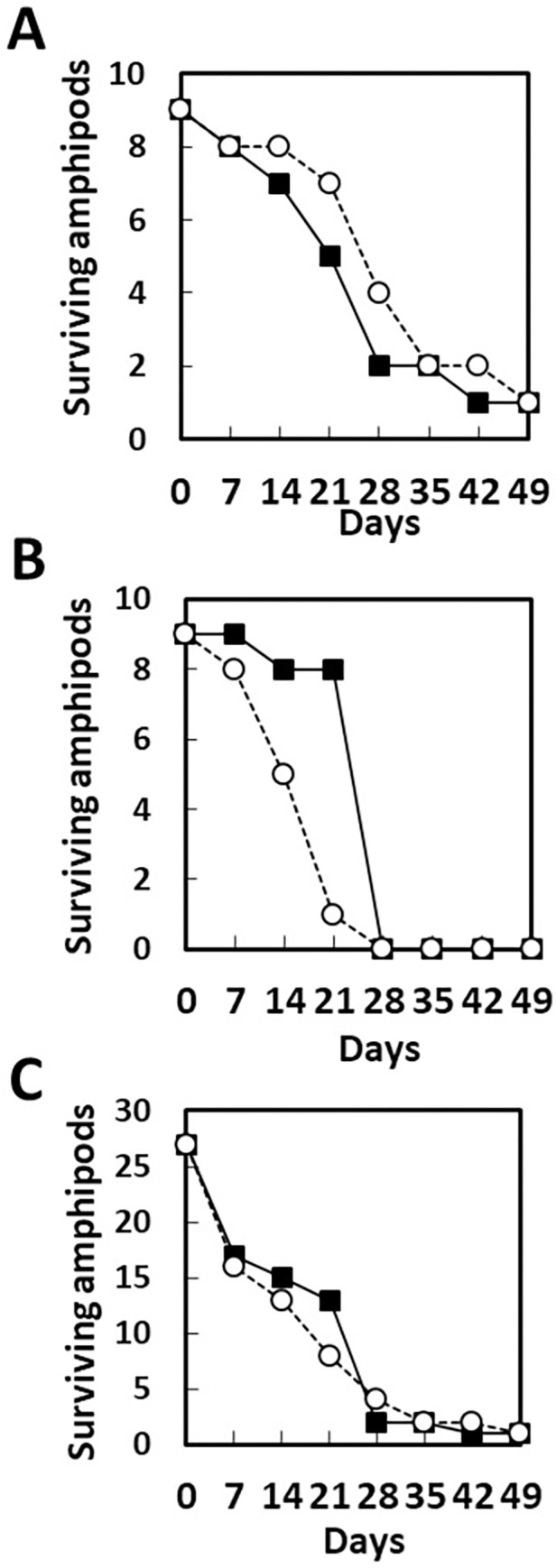
First (A), second (B) and third (C) life span experiments. Amphipods were washed and released into beakers with (dotted line, open circles) or without (line, closed squares) antibiotics. The numbers of surviving amphipods were counted.

We anticipated that talitrid amphipods feeding on algae would die or their feeding on algae would be slowed after the gut bacteria were killed by the antibiotics, if these bacteria were essential for assimilation of algae. However, the amphipods fed on algae or paper at about the same rate, irrespective of the presence or absence of antibiotics (S7 Fig in S1 File). To examine the effect of antibiotics on life span, the amphipods were housed with or without antibiotics for a long period. A count of the number of surviving amphipods indicated no effect of the antibiotics on the life span (Fig 5).

To identify the microbes in the gut of amphipods housed in sterilized condition with or without antibiotics, DNA was isolated from the guts of amphipods housed for 14 days (1 amphipod from condition H [algae as feed, without antibiotics], 5 from condition I [algae as feed, with antibiotics], and 4 from condition J [paper as feed, with antibiotics]) and metagenomic analysis was conducted using 16S and ITS2 data obtained from these samples. As shown in Figs 3 and 4 (conditions H–J), both 16S and ITS2 data showed that intervention with antibiotics completely killed *Vibrio* sp. and had significant effects on the composition of the flora in the gut, with elimination of the variation in the guts of amphipods housed without antibiotics.

For 16S data, antibiotics with algae as feed had a composition consisting of unassigned bacteria, and genera *Echinicola* and *Pseudomonas*, while antibiotics with paper as feed gave a composition of genera *Rhodococcus*, *Ochrobactrum*, and *Pseudomonas* (Fig 3 and S1 Table). We note that these genera were also sometimes detected in the absence of antibiotics, although at very low frequency, indicating that these genera flourished after antibiotic killing of bacteria such as *Vibrio* sp., *Paracoccus* sp., and *Pseudoalteromonas* sp., which had prospered in the absence of antibiotics.

For ITS2 data, antibiotics with algae as feed gave a composition of genera *Acremonium* (Nos. 37–41), *Sarocladium* (Nos. 37, 39), unidentified fungi*1 (No. 37), and *Nectriaceae* (No. 40), while antibiotics with paper as feed gave a composition of genera *Gibellulopsis* (Nos. 42–45), *Sarocladium* (Nos. 43–45), *Acremonium* (No. 45), and unidentified fungi*1 (No. 44) (Fig 4 and S2 Table). Beta diversity analysis using weighted UniFrac distances for 16S and ITS2 data indicated that data from conditions H, I, and J clustered separately from each other (S8 Fig in S1 File). The antibiotics seemed not to affect the fungi directly, which suggests that antibiotics have indirect effects on the fungi by causing significant changes in the gut bacterial composition.

We attempted to isolate microbes from guts of talitrid amphipods housed with algae and antibiotics (condition I, Table 2). Although *Echinicola* and *Pseudomonas* are major genera in the guts of these amphipods (Fig 3, S1 Table), we failed to isolate these genera. Instead, we isolated two bacteria using marine broth, which were identified as *Aeromonas bivalvium* and *Stenotrophomonas maltophilia* (Table 4). *A*. *bivalvium* was not detected in metagenomic analysis, while *S*. *maltophilia* was detected at higher frequency in the presence of antibiotics compared to that in the absence of antibiotics (S1 Table). Alginate-assimilation by these two bacteria were not confirmed due to the absence of growth in NTA or TA medium (S9A Fig in S1 File). The alginate-degrading activity of the two bacteria was also unclear: both bacteria showed a halo in Gram’s iodine staining, but alginate degradation was not found in TLC analysis (S9B Fig in S1 File).

**Table 4:** Bacteria isolated from guts of amphipods housed with antibiotics.

Strains	Top hit species ^a^	Top hit strain ^b^	Similarity (%) ^c^	Size of query (b)
SK6610 ^d^	*Aeromonas bivalvium*	CECT 7113	99.64	1,407
SK6611^d^	*Stenotrophomonas maltophilia*	MTCC 434	99.84	1,286

^a, b, c^ The query sequence shows the highest hit for species^a^ and strain^b^ with similarity^c^.

^d^ SK6610 and SK6611 were isolated using Marine broth, and SK6611 was isolated in an enrichment culture.

### Role of *Vibrio* sp. in the gut of talitrid amphipods

Animal glycoside hydrolase 7 (GH7) cellobiohydrolase in the marine wood borer *Limnoria quadripunctata* (LqCel7B) has recently been characterized and shown to be secreted in the gut of *L*. *quadripunctata* for wood degradation [30]. A Blastp search using the primary structure of LqCel7B gave three homologs in amphipods, including the talitrid amphipod *Trinorchestia longiramus* (S3 Table), suggesting that GH7 cellobiohydrolase functions in the gut of talitrid amphipods. This enzyme may digest brown macroalgae for assimilation and to enhance access of microbes, especially *Vibrio* sp., to alginate.

Microbes in the gut of talitrid amphipods may have effects on the hosts. Abdelrhman *et al*. [8] detected glycosyl hydrolase family 48 gene, which is a measure of cellulolytic bacterial diversity in environmental samples [31], in the gut microbiota of five species of supralittoral talitrid amphipods, suggesting involvement of gut microbiota in carbon source assimilation. In the current study, *Vibrio* sp. was detected at high frequency in the gut, and killing of bacteria such as *Vibrio* sp., *Paracoccus* sp., and *Pseudoalteromonas* sp. with antibiotics had no effect on the life span of amphipods (Fig 5). These results suggest that microbes that were killed by antibiotics, including *Vibrio* sp. and *Pseudoalteromonas* sp., are not essential for assimilation of brown macroalgae in these amphipods. The possibility remains that microbes that flourished after antibiotic killing of bacteria such as *Vibrio* sp. may have roles in assimilation of brown macroalgae.

## Supporting information

S1 File Supplementary Figs S1-S9. (DOCX)

S1 Table Frequency of the genera of bacteria in each sample. (XLSX)

S2 Table Frequency of the genera of fungi in each sample. (XLSX)

S3 Table Cellobiohydrolase homologs found in amphipods. (DOCX)
