# Correction: Bacterial and fungal gut microbiota of supralittoral talitrid amphipods feeding on brown macroalgae and paper

**DOI:** 10.1371/journal.pone.0315421

**Published:** 2024-12-05

**Authors:** The PLOS ONE Staff

The Data Availability statement for this paper is incorrect. The correct statement is: All sequences generated in the study are publicly available. Metagenomic data have been deposited in the DDBJ Sequence Read Archive database under accession numbers: DRA014268 (16S), DRA014304 (ITS2), and DRA014305 (18S) (first set); and DRA014306 (16S, ITS2, and 18S) (second set). Partial 16S rRNA sequences have been placed in the DDBJ database: LC722366 (SK6405), LC722367 (SK6416), LC722368 (SK6401), LC722369 (SK6402), LC722370 (SK6376), LC722371 (SK6421), LC722372 (SK6418), LC722373 (SK6422), LC722374 (SK6371), LC722375 (SK6415), LC737961 (SK6610), and LC737962 (SK6611). The publisher apologizes for the error.
