# Supplementary material for: Bacterial and fungal gut microbiota of supralittoral talitrid amphipods feeding on brown macroalgae and paper
Source: PLoS One. 2022 Dec 30;17(12):e0279834. doi: 10.1371/journal.pone.0279834 (PMC9803094; doi:10.1371/journal.pone.0279834)
Supplement: S1 File — (DOCX) [file pone.0279834.s001.docx]

A


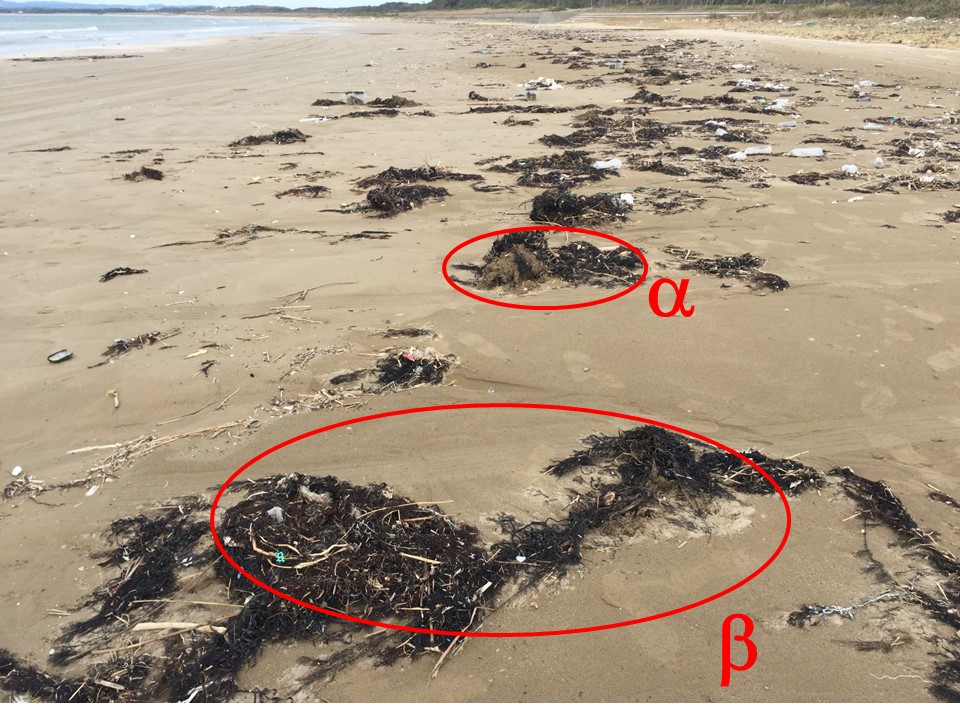


B


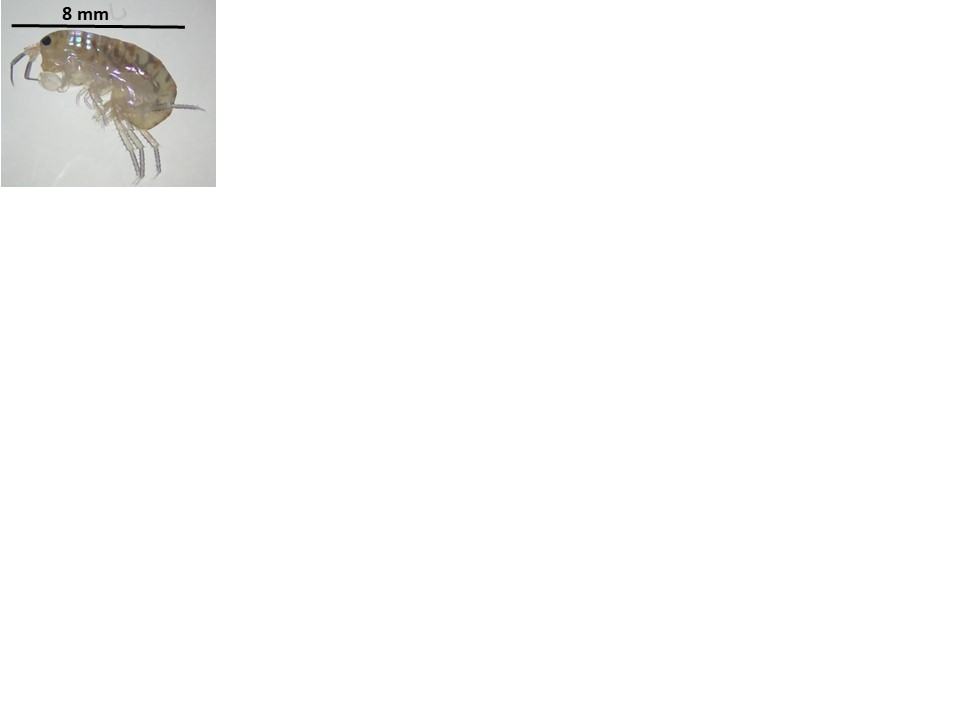


**Supplementary Fig. S1.** **Brown macroalgae (gulfweed) and a beach hopper (talitrid amphipod).** A: Brown macroalgae (gulfweed) stranded on the Shibagaki coast. “α” and “β” indicate stranded macroalgae from which amphipods were collected. The photograph was taken on Oct. 17, 2019. B: A beach hopper (talitrid amphipod).


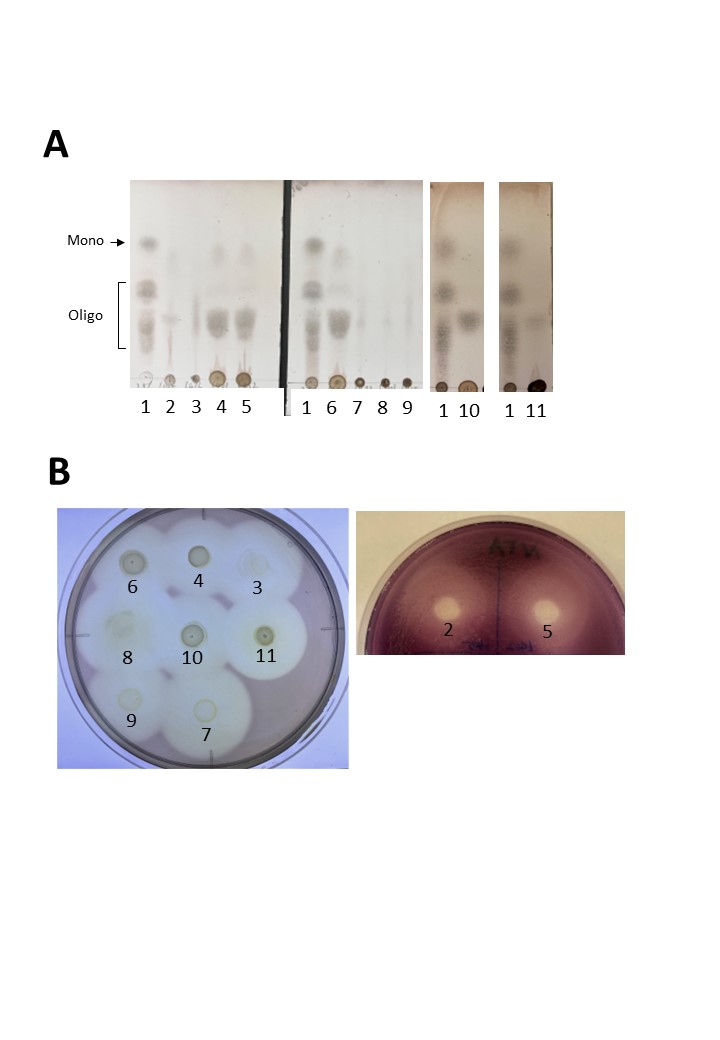


**Supplementary Fig. S2. Alginate-degrading activity.** A: Activity was visualized using TLC, as described in the Materials and Methods. Lane: 1, Mixtures of DEH (monouronate) and oligoalginates. Lanes 2-11: Supernatants of cultures of the following strains: 2, SK6405, 3, SK6416, 4, SK6401, 5, SK6402, 6, SK6376, 7, SK6422, 8, SK6371, 9, SK6415, 10, SK6418, and 11, SK6421. The details of these strains are listed in Table 3. B: Activity was visualized with Gram’s iodine, as described in the Materials and Methods. Numbers correspond to the strains in A. The presence of a clearance zone indicates that the alginate is degraded [12].


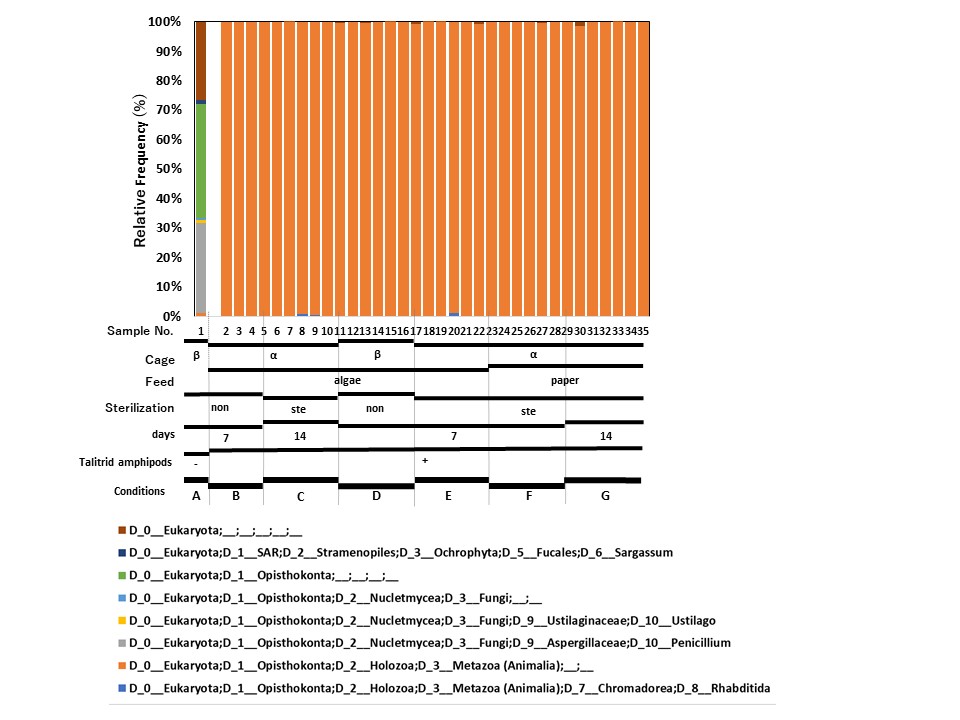


**Supplementary Fig. S3. Bar plot of relative frequencies in the 18S data at the genus level.**


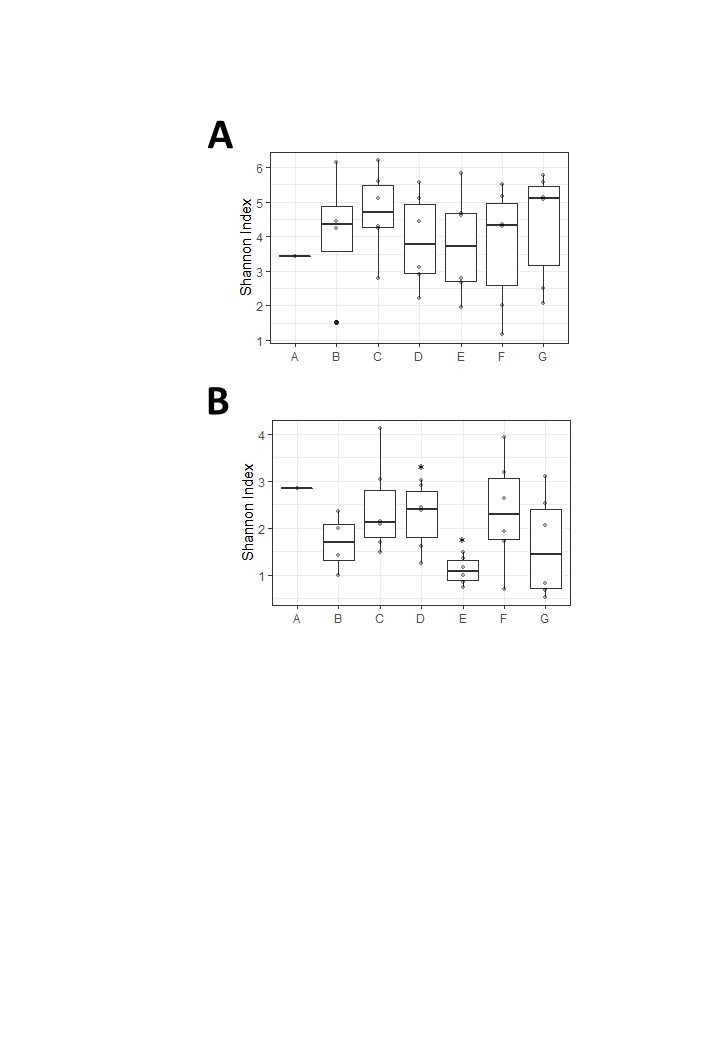


**Supplementary Fig. S4. Shannon alpha-diversity metric for 16S (A) and ITS (B) data.**


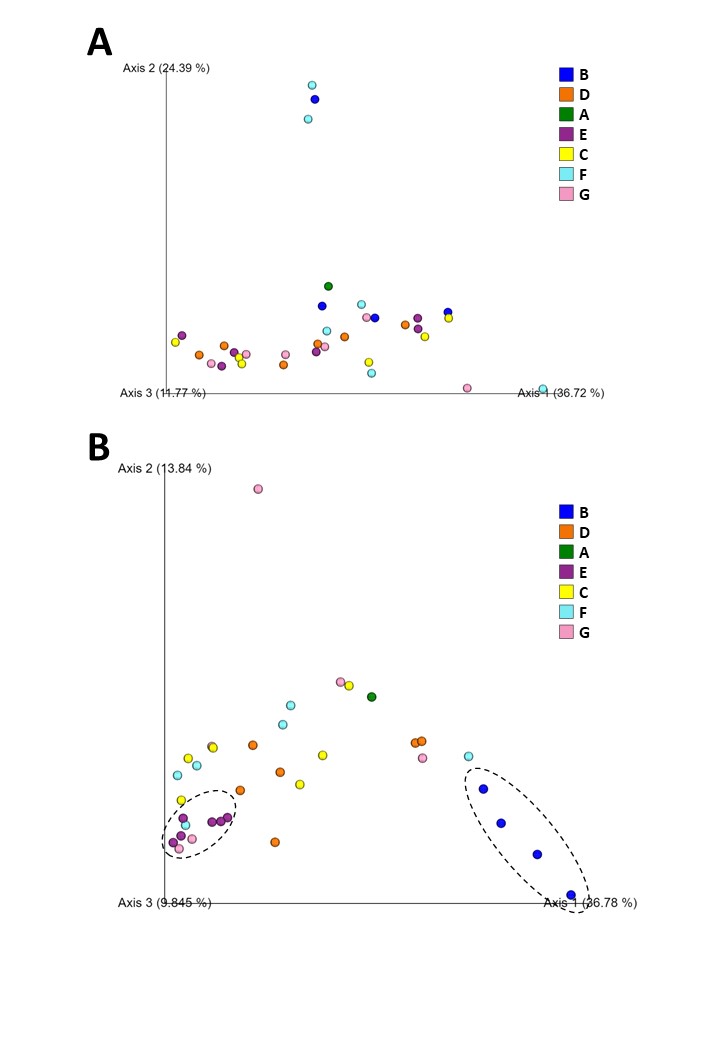


**Supplementary Fig. S5. Beta diversity analysis using weighted UniFrac distances for 16S (A) and ITS (B) data.**


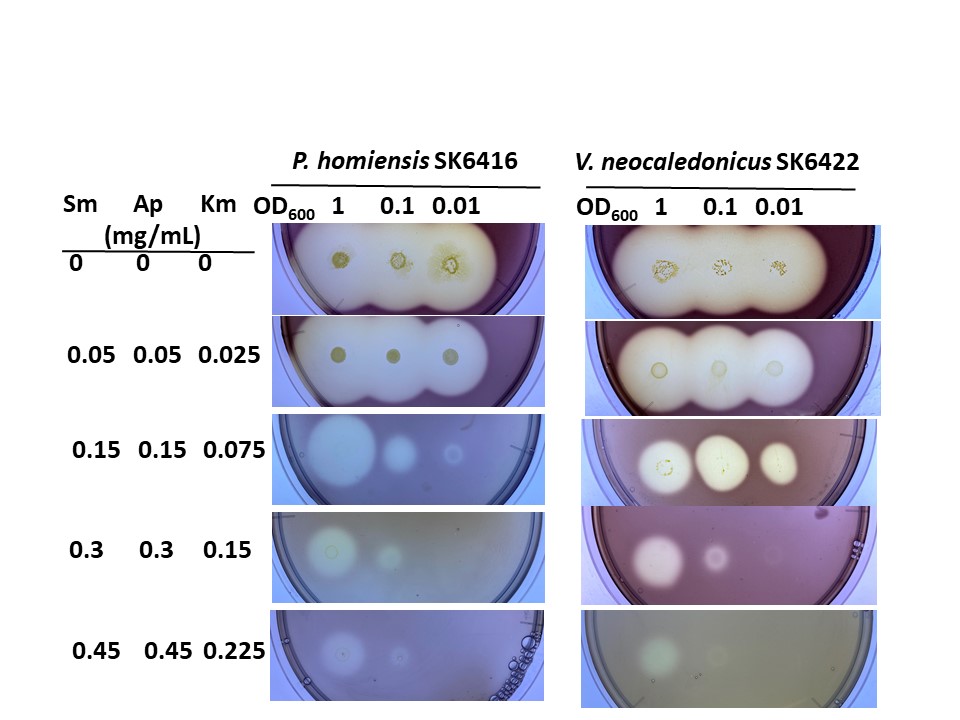


**Supplementary Fig. S6. Determination of suitable concentrations of antibiotics.** The indicated strains were grown in the presence of the indicated concentrations of antibiotics (Sm, streptomycin; Ap, ampicillin; Km, kanamycin). as described in the Materials and Methods.


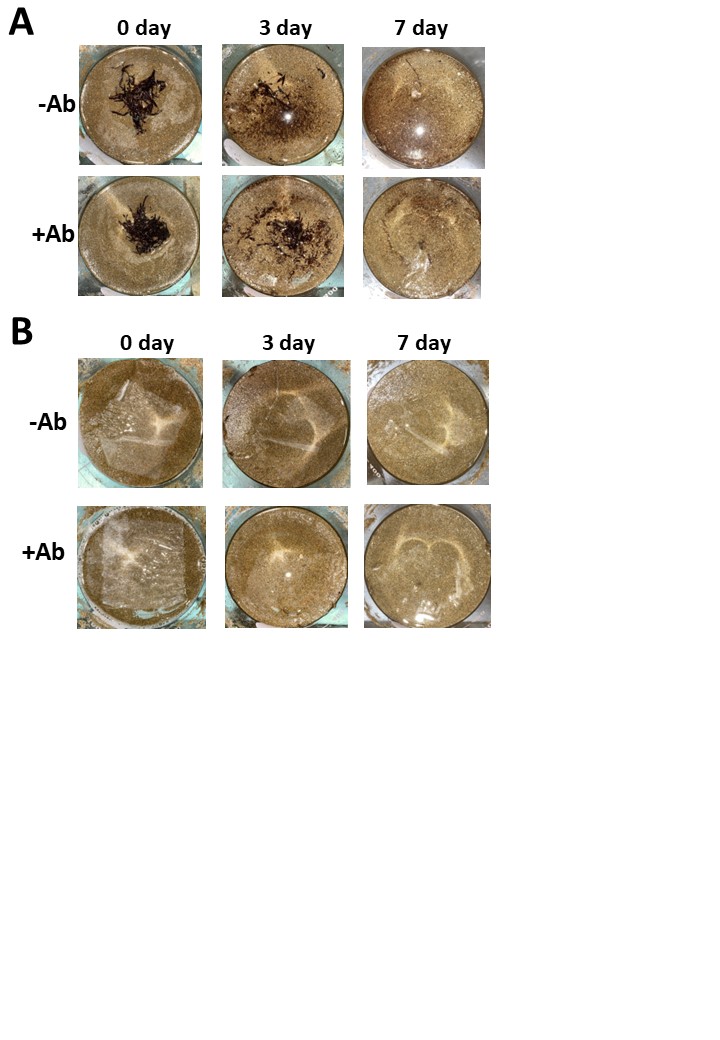


**Supplementary Fig. S7. Antibiotics had no effect on the feeding rate of talitrid amphipods.** Stranded brown macroalgae (A) and paper (B) with 10 individual amphipods in the absence (-Ab) or presence (+Ab) of antibiotics were observed for the indicated periods under sterilized conditions.


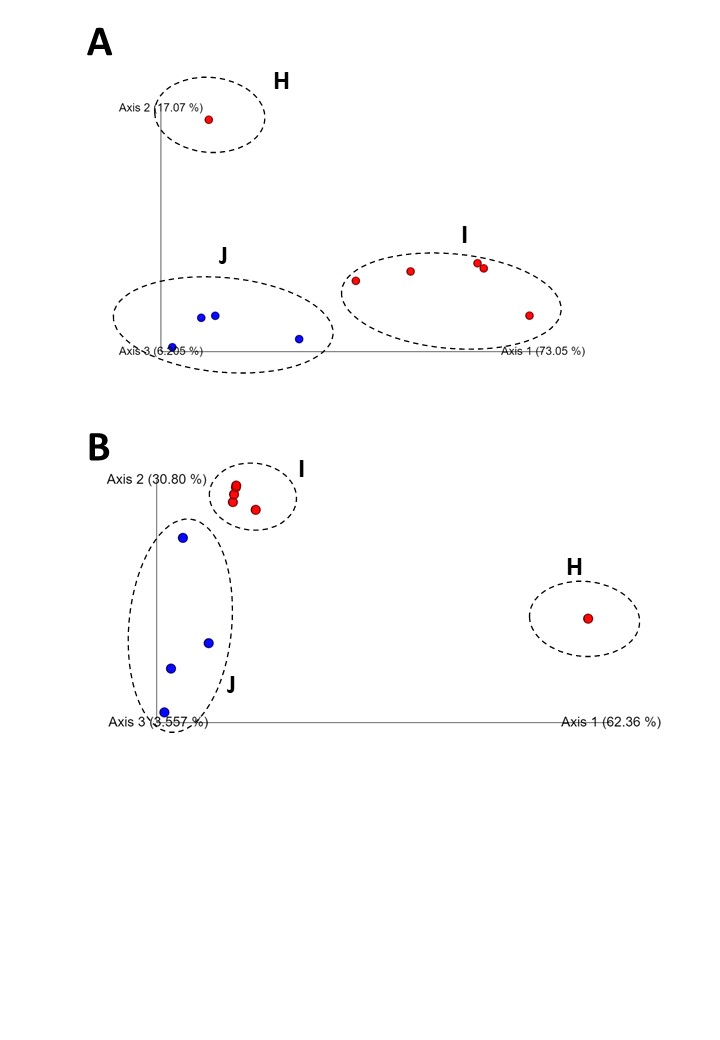


**Supplementary Fig. S8. Beta diversity analysis using weighted UniFrac distances for 16S (A) and ITS (B) data.**

**
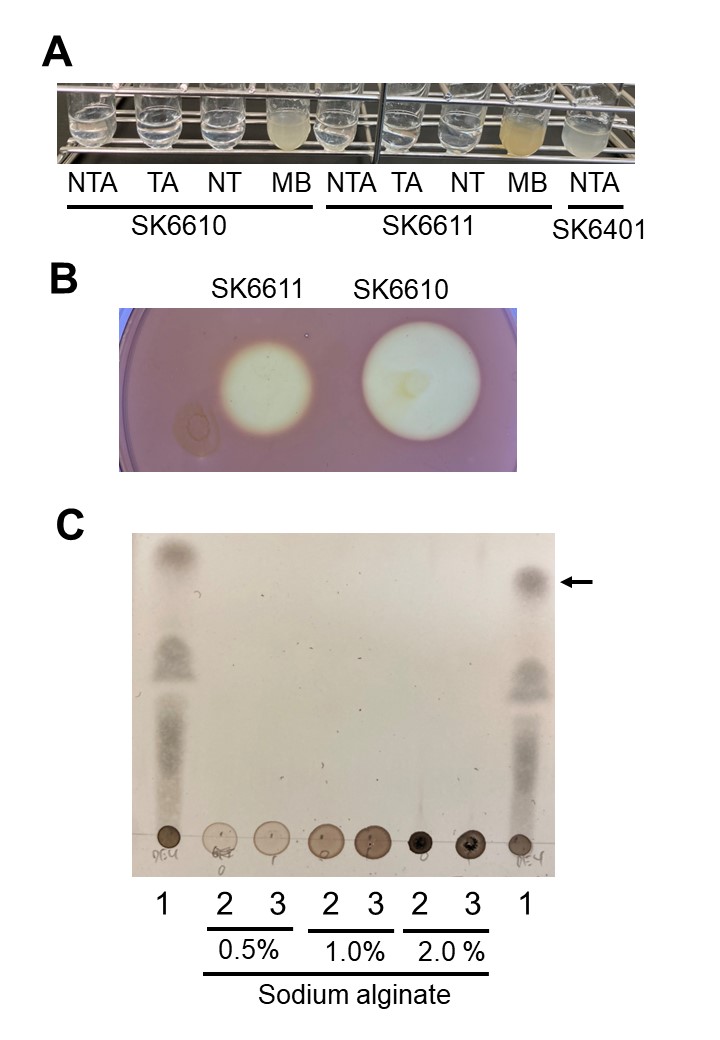
**

**Supplementary Fig. S9. Growth and alginate-degradation of SK6610 and SK6611 strains.** A: SK6610 and SK6611 strains were precultured in liquid marine broth medium, washed twice with NT medium, and suspended in NT medium to reach OD_600_ of 1.0. The suspension (50 µL) was inoculated into 1.0 mL of the indicated liquid media and cultivated for 2 days. MB: Marine broth with 1.0% sodium alginate. As a control, the SK6401 strain was precultured in liquid NTA medium and treated as above. B: SK6610 and SK6611 strains were precultured in solid marine broth medium and suspended in NT medium to reach OD_600_ of 1.0. The suspension (5 µL) was spotted onto NTA solid medium, cultivated for 3 days, and stained with Gram’s iodine. C: SK6610 and SK6611 strains were cultured as in A for 2 days in MB liquid medium containing the indicated concentration of sodium alginate. The supernatant was analyzed by TLC as described in the Materials and Methods. Lanes: 1, mixture of DEH (monouronate) and oligoalginates; 2, SK6610; 3, SK6611. The arrow indicates DEH.
