# Supplementary material for: Bacterial and fungal gut microbiota of supralittoral talitrid amphipods feeding on brown macroalgae and paper
Source: PLoS One. 2022 Dec 30;17(12):e0279834. doi: 10.1371/journal.pone.0279834 (PMC9803094; doi:10.1371/journal.pone.0279834)
Supplement: S3 Table — (DOCX) [file pone.0279834.s004.docx]

**Supplementary Table S3.** Cellobiohydrolase homologs found in amphipods

| Organism | ID | Length | e-value | Identity  (%) | Positive  (%) | Gap  (%) |
| --- | --- | --- | --- | --- | --- | --- |
| *Hyalella azteca* ^a^ | XP_018027108.1 | 455 | 0.0 | 298/442  (67) | 339/442  (76) | 11/442  (2) |
| *Chelura terebrans* ^b^ | AGM37865.1 | 463 | 0.0 | 289/441  (66) | 343/441  (77) | 10/441  (2) |
| *Trinorchestia longiramus* ^c^ | KAF2364826.1 | 440 | 0.0 | 280/441  (63) | 331/441  (75) | 12/441  (2) |

^a^ [*Hyalellidae*](https://www.ncbi.nlm.nih.gov/Taxonomy/Browser/wwwtax.cgi?mode=Undef&id=199477&lvl=3&keep=1&srchmode=1&unlock) family

^b^ [*Cheluridae*](https://www.ncbi.nlm.nih.gov/Taxonomy/Browser/wwwtax.cgi?mode=Undef&id=1336363&lvl=3&keep=1&srchmode=1&unlock) family

^c^ [*Talitridae*](https://www.ncbi.nlm.nih.gov/Taxonomy/Browser/wwwtax.cgi?mode=Undef&id=92169&lvl=3&keep=1&srchmode=1&unlock) family
